# Bacteria and fungi of the lung: allies or enemies?

**DOI:** 10.3389/fphar.2024.1497173

**Published:** 2024-11-08

**Authors:** Enrico Garaci, Marilena Pariano, Emilia Nunzi, Claudio Costantini, Marina Maria Bellet, Cinzia Antognelli, Matteo Antonio Russo, Luigina Romani

**Affiliations:** ^1^ San Raffaele Research Center, Sulmona, L’Aquila, Italy; ^2^ Department of Medicine and Surgery, University of Perugia, Perugia, Italy; ^3^ IRCCS San Raffaele Roma, Rome, Italy

**Keywords:** respiratory bacteriome, respiratory mycobiome, bacterial-fungal interactions, immunity, metabolism

## Abstract

Moving from the earlier periods in which the lungs were believed to represent sterile environments, our knowledge on the lung microbiota has dramatically increased, from the first descriptions of the microbial communities inhabiting the healthy lungs and the definition of the ecological rules that regulate its composition, to the identification of the changes that occur in pathological conditions. Despite the limitations of lung as a microbiome reservoir due to the low microbial biomass and abundance, defining its microbial composition and function in the upper and lower airways may help understanding the impact on local homeostasis and its disruption in lung diseases. In particular, the understanding of the metabolic and immune significance of microbes, their presence or lack thereof, in health and disease states could be valuable in development of novel druggable targets in disease treatments. Next-generation sequencing has identified intricate inter-microbe association networks that comprise true mutualistic or antagonistic direct or indirect relationships in the respiratory tract. In this review, the tripartite interaction of bacteria, fungi and the mammalian host is addressed to provide an integrated view of the microbial-host cross-talk in lung health and diseases from an immune and metabolic perspective.

## 1 Introduction

The last 15 years have witnessed a boost of studies on the respiratory microbiota, i.e., the collection of microbial communities that inhabit the surface of the upper and lower respiratory tracts. We have come to the point where the lung microbiome characterizes with a low biomass in healthy conditions, containing 10^3^ to 10^5^ bacteria per gram of tissue as compared to a density of 10^11^ to 10^12^ bacteria per gram in the large intestine, as a result of the balance between the influx of microbes from the upper respiratory tract and their elimination by means of mechanical and immune-mediated mechanisms, including cough, mucociliary clearance and phagocytosis by alveolar macrophages (Dickson et al., 2016; Wypych et al., 2019). The respiratory microbiome is vital for immune training, organogenesis and the maintenance of immune tolerance. Increasing evidence supports the existence of a window of opportunity early in life, during which adequate microbiota sensing is essential for immune maturation and consecutive respiratory health. In turn, the immune system controls the lung microbial biomass via a balanced immune tolerance and immune clearance mechanisms (Wypych et al., 2019; Park et al., 2024; Natalini et al., 2023; Lipinksi et al., 2024). Thus, the alterations of the structural and functional properties of the lung may favor the local growth of microbial communities, whose identity appears to be related to the underlying pathological condition and usually associated with uncontrolled inflammation (Natalini et al., 2023; Lipinksi et al., 2024).

Next-generation sequencing has identified intricate inter-microbe association networks that comprise true mutualistic, commensal or antagonistic direct or indirect relationships. Alternatively, microbial co-occurrence could be driven by host and environmental factors. In this review, starting with a brief overview of the bacteriome and mycobiome of the respiratory tract, the tripartite interaction of bacteria, fungi and the mammalian host is addressed to provide an integrated view of the microbial-host cross-talk in lung health and disease from an immune and metabolic perspective.

## 2 The respiratory bacteriome

Although the dynamics of upper and lower airways in health and disease is a research topic continuously evolving, the upper respiratory tracts (URT) (nasal cavity, mouth, pharynx and upper bronchus) have microbiota resembling the oral cavity, thus heavily influenced by environmental factors. The mode of delivery, type of infant feeding, diet and antibiotics play a key role in the development of the microbiota of the URT which can be considered an important predictor of the frequency of respiratory infections in children (Teo et al., 2018) and may continue to play a role in respiratory health and disease later in life. In contrast, the lower respiratory tract (LRT) has unique microbial signatures, apparently mildly influenced by microbiota from other mucosal surfaces, although the source and resilience of lower airway microbiome is still debated (Natalini et al., 2023; Lipinksi et al., 2024; Dickson et al., 2013). Despite the uncertainties regarding the definition of the basic composition of “healthy” lung microbiome, Actinobacteria and Firmicutes phyla, with smaller proportions of species from the Proteobacteria and Bacteroidetes, dominate in the upper lung, while Bacteroidetes and Firmicutes, with smaller proportions of species from the Proteobacteria, dominate in the lower tract (Charlson et al., 2011; Man et al., 2017). *Corynebacterium* spp., along with other lipophilic skin colonizers, such as *Cutibacterium* and *Staphylococcus* spp., commonly dominate in the anterior nares, *Moraxella*, *Dolosigranulum* and *Streptococcus* spp. dominate the nasal mucosa while *Dolosigranulum*, *Haemophilus* and *Streptococcus* spp. are frequent colonizers of the nasopharynx along with *Moraxella*, *Corynebacterium*, and *Staphylococcus* spp.*. D. pigrum* can produce lactic acid giving it the potential to lower the pH of the local environment which may select for *Corynebacterium* spp. growth, potentially explaining their co-occurrence within the URT (de Steenhuijsen Piters et al., 2015). It has been suggested that *Dolosigranulum* and *Corynebacterium* spp. act as ‘keystone species’, for their association with respiratory health and resistance to *Streptococcus* pneumonia infection (Man et al., 2017). In healthy children and adults, a unique microbial community in the LRT was found to contain many of the bacteria that are common to the URT including Firmicutes (*Staphylococcus*, *Streptococcus* and *Veillonella* spp.), Bacteriodetes (*Prevotella* spp.) and Proteobacteria (*Porphyromonas*, *Moraxella* and *Haemophilus* spp.) (Man et al., 2017). The URT is colonized by transient microorganisms which could possibly prevent potential pathogens from overgrowing and disseminating towards the lungs, thereby functioning as gatekeepers to respiratory health through different mechanisms of colonization resistance such as the production of antimicrobial peptides, niche and nutrient completion, mucociliary and immune clearance and alteration of microbe physiology (Natalini et al., 2023; Man et al., 2017; Mendez et al., 2019). This may occur against both airborne pathogens and pathobionts, namely *Streptococcus pneumoniae*, *Staphylococcus aureus*, *Moraxella catarrhalis* and *Haemophilus influenza*.

Several reports have implicated dysbiosis in the pathogenesis of many lung diseases, such as the increased abundance of: i) *Pseudomonas*, *Staphylococcus* and *Burkholderia* spp. in Cystic fibrosis (CF) (Blanchard and Waters, 2019; Cox et al., 2010); ii) *Haemophilus*, *Streptococcus*, *Moraxella*, *Pseudomonas*, *Neisseiria* spp. and gram-negative enteric bacteria in chronic obstructive pulmonary disease (COPD) and bronchiectasiae (Mac et al., 2024; Wang et al., 2021); iii) *Prevotella* and *Veillonella* spp. and *Escherichia coli* in idiopathic pulmonary fibrosis (Amati et al., 2022); iv) Comamonadaceae, Sphingomonadaceae and Oxalobacteraceae in bronchial hypersensitivity (Huang et al., 2011) and v) the variable microbial composition in allergy, depending on the asthma endotypes. The neutrophilic-Th2-low asthma was associated with an increase in pro-inflammatory Proteobacteria, including *Moraxella*, *Haemophilus* or *Neisseria* spp. and a decrease in genera that belong to the phyla Firmicutes, Actinobacteria and Saccharibacteria (Sharma et al., 2019), while eosinophilic asthma can correlate with the abundance of Firmicutes (e.g., *Streptococcus*), Actinomycetaceae and Enterobacteriaceae (Sharma et al., 2019; Loverdos et al., 2019). Similarly, evidence from studies of lung-transplant recipients suggests a link among neutrophilia, inflammatory macrophages and the emergence of inflammatory bacteria belonging to the Proteobacteria phylum (McGinniss et al., 2021). The finding of an increased abundance of *Staphylococcus* and *Enterococcus* spp., *Klebsiella pneumoniae*, as well as fungi like *Candida* spp. or the mold *Rhizopus*, in COVID-19 patients (Merenstein et al., 2022) is consistent with the complex and dynamic interaction of the local microbiota with pneumonia in which the enrichment of the lower respiratory microbiota and oral bacteria is correlated with subclinical inflammation that ignites a vicious cycle of homeostatic disturbance and increased inflammation (Dickson et al., 2014). The clinical relevance of the microbiota in the initiation, progression, and treatment outcomes of lung cancer has recently been discussed (Natalini et al., 2023; Bou Zerdan et al., 2022).

Clinical evidence suggests that restoration of gut microbiome positively affects lung microbial homeostasis and, by association, lung immune homeostasis (Mendez et al., 2019), a finding consistent with the association of pulmonary dysfunction and diseases with inflammatory bowel diseases (Zhou et al., 2021) but also *vice versa* (Rutten et al., 2014; Marsland et al., 2015), thus suggesting a reciprocal influence of the microbiota in the respiratory and intestinal tracts. For instance, the shift from Gammaproteobacteria and Firmicutes toward Bacteroidetes in the gut during the first 2 weeks of life was found to be associated with the accumulation of a PD-L1-dependent T-regulatory cells (Tregs) that promote tolerance to allergen challenge via the supply of microbial metabolites (Gollwitzer et al., 2014; Depner et al., 2020). Studies have indeed implicated the host sensing of local and intestinal microbial metabolites as a mechanism underlying the interventional cross-talk between the two ecosystems (Budden et al., 2017). For example, a significant reduction of the microbial metabolites including the short chain fatty acids (SCFAs) acetate, butyrate and propionate, in the feces from patients with bronchial asthma was observed when compared with healthy controls (Ivashkin et al., 2019) and *Faecalibacterium prausnitzii* and *Akkermansia muciniphila* have been reported to suppress the inflammatory responses in pediatric allergic asthma (Demirci et al., 2019). Intestinal dysbiosis, with reduction of anaerobes and their SCFAs metabolites in parallel with the expansion of potential pathogens, has been associated with adverse outcome in critical illness (Cho et al., 2024). The intensive use of broad-spectrum antibiotics and other pharmacological interventions, changes in nutritional intake as well as the presence of multi-organ morbidities, all work in concert to alter the composition and function of the gut microbiota and impair colonization resistance mechanisms (Cho et al., 2024). As a consequence, hospital-acquired infections, most commonly in the lung or disseminated, often develops with severe consequences for the patients. While translocation of gut-derived microbes in the lung may drive nosocomial pneumonia, dysbiosis of the airway microbiota may similarly play a role (Cho et al., 2024), pointing to the need for careful dissection of local and distal gut-lung interactions. Although much has still to be done in terms of mechanism of action of probiotics and the formulation approaches in terms of pharmaceutical technology, some studies have demonstrated the effectiveness of probiotics in lung diseases and infections (Wypych et al., 2019; Yuksel et al., 2023; Xavier-Santos et al., 2022).

## 3 The respiratory mycobiome

**FIGURE 1:**
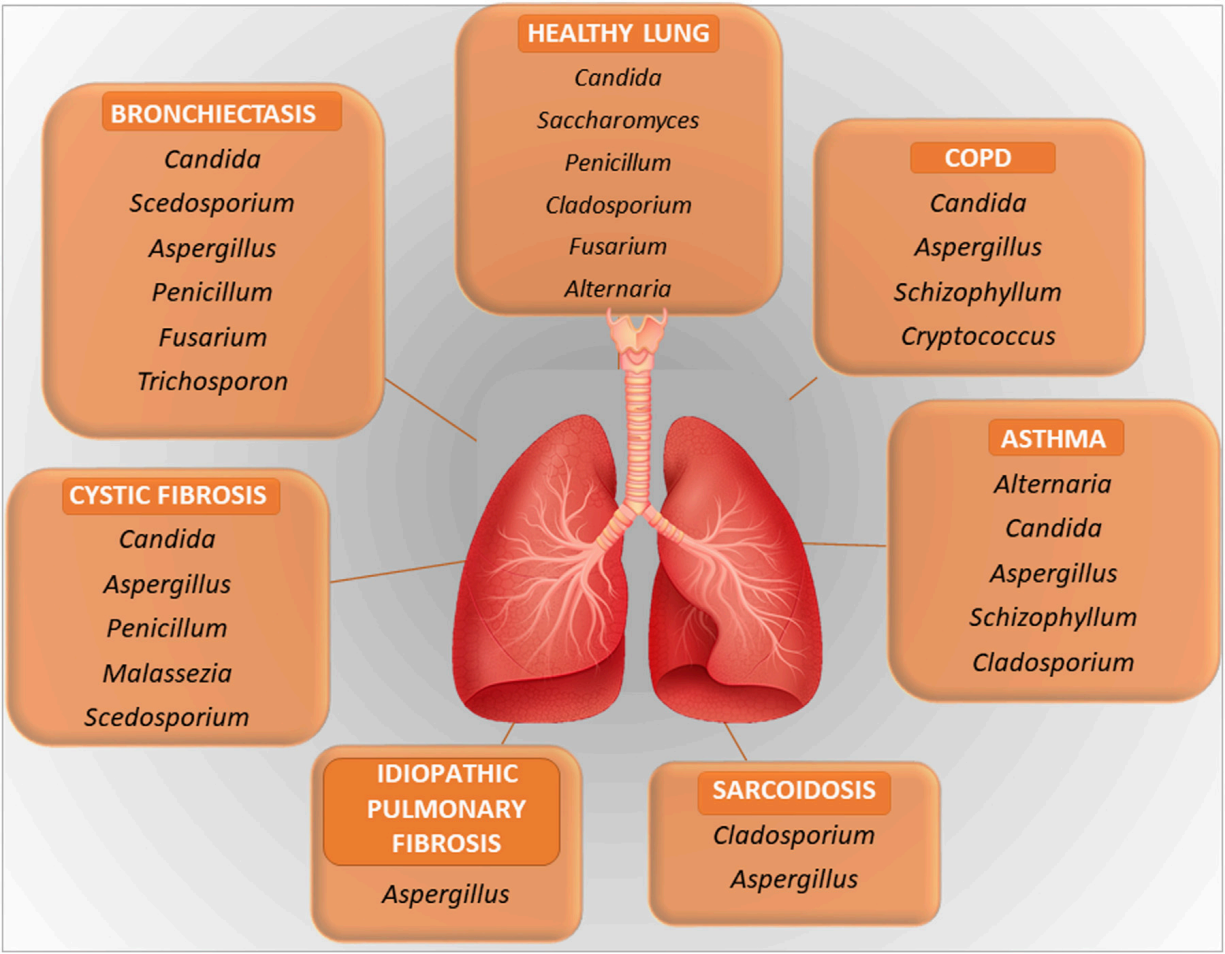
A figure illustrating the key microbial components in healthy lung and in lung diseases. Further explained in the text.

The relevance of the fungal component of the microbiota, or mycobiome, in the respiratory tract is increasingly being recognized in health and diseases (Nguyen et al., 2015). Although the size of the respiratory mycobiome is unknown, the gut and skin mycobiomes are approximated to comprise 0.1% and 3.9%, respectively, of the total microbiome in their corresponding niches (Cui et al., 2013). Like bacteria, fungi are directly involved in regulating the pulmonary environment, the local immune response and may contribute to decreased lung function and disease progression (de Dios Caballero et al., 2022; Li et al., 2024). In addition, fungi influence bacterial behavior through different interactions, resulting in positive or negative interactions between members of the lung microbiome, as outlined in the next paragraph. The fungal communities inhabiting the LRT follows the same ecological rules of bacteria with aspiration from the URT representing a major immigration route. In addition, the lungs may be exposed to the spores of fungi that transiently populates the fungal community. This is a typical immigration route for filamentous fungi. The healthy URT has a mycobiota that includes Ascomycota and Basidiomycota with *Candida*, *Saccharomyces*, *Penicillium*, *Alternaria*, *Cladosporium*, and *Fusarium* spp. being the most common genera in the oral and nasal cavities (Cui et al., 2015; Palmieri et al., 2022) (Figure 1). The presence of *Fusarium* and *Cryptococcus* in the lungs but not in the oral cavity of healthy individuals suggests that the fungal communities harbored in the lower airways are distinct from those harbored in the upper airways.

In pathological conditions, the mycobiome may be subjected to quantitative and qualitative changes with a disease-specific pattern, similarly to the bacterial counterpart and to which fungal dysbiosis of the gut could also contribute (Sey and Warris, 2024). For instance, the clinical manifestations in COPD are associated with alterations in the composition of the mycobiome, with a role for *Aspergillus* in exacerbation and allergic inflammation, although other fungi may also play a role (Li et al., 2024). Another example is represented by CF in which, beside the most common respiratory pathogens, *Pseudomonas aeruginosa* and *S. aureus*, *Aspergillus fumigatus* is emerging as a critical player in pulmonary exacerbations. *Aspergillus* may be the cause of several clinical manifestations in CF, including bronchitis, sensitization and allergic bronchopulmonary aspergillosis (Figure 1). Pointing to the importance of the inter-kingdom cross-talk in the lung, as further discussed below, *P. aeruginosa* often precedes *A. fumigatus* infection. Despite the recognized role of *Aspergillus* in CF, *Candida* species and additional fungal species such as *Cladosporium, Scedosporium* and *Exophiala* spp. are also found in the LRT in CF (Tracy and Moss, 2018; Krause et al., 2016). Inhaled fungi, such as *Aspergillus*, *Cladosporium* and *Penicillium* spp, can be predominant triggers of asthma exacerbations (Sharpe et al., 2015). Along with bacteria, fungi may also possibly associate with nonresolving acute respiratory distress syndrome in pneumonia (Kullberg et al., 2022). A recent potential role attributed to fungi is in cancer (Dohlman et al., 2022). Analysis of lung cancer mycobiome revealed association of *Malassezia* and *Blastomyces* with tumor tissues (Dohlman et al., 2022). More recently, the intratumor fungus *Akkermansia sydowii* promoted the expansion and activation of myeloid-derived suppressor cells that, in turn, suppressed cytotoxic T lymphocytes and promoted tumor escape (Liu et al., 2023). Very interestingly, the accelerated tumor growth by *A. sydowii* was prevented by aerosolized inhaled Amphotericin B, indicating that *A. sydowii* was part of the lung mycobiome and not of gut origin. This was in line with the presence of *A. sydowii* in exhaled breath condensate and in lung tissues of patients. Therefore, fungal commensals in the respiratory tract may be implicated in cancer initiation and progression, with obvious repercussions in cancer research and future therapeutic approaches (Liu et al., 2024).

## 4 Bacterial-fungal interactions in the respiratory tract

**FIGURE 2:**
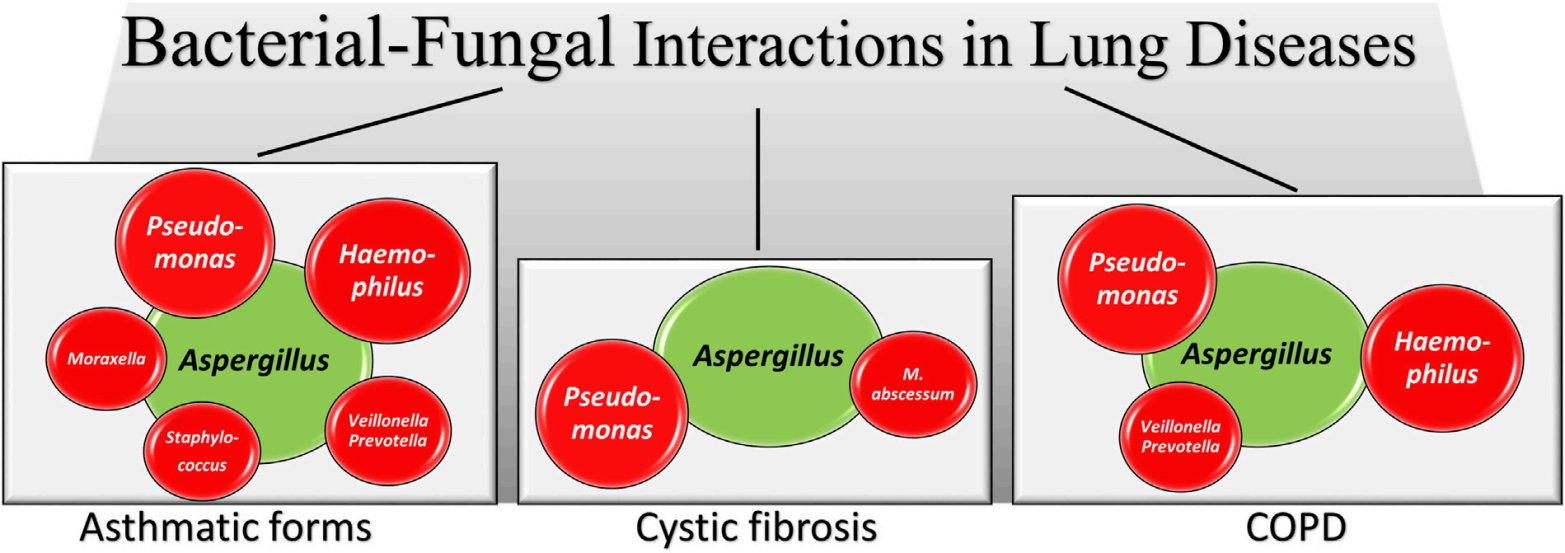
A schematic illustration of *Aspergillus*-bacteria interactions in lung diseases. Irrespective of the antagonistic or synergistic nature of the interactions (fully described in the text), the figures shows how *Aspergillus* (green) establishes interactions with different bacterial species (red, size reflects the relative importance) in different lung pathologies. In the asthmatic forms data are from asthma, allergic bronchopulmonary aspergillosis, mild asthma, severe asthma and severe asthma with fungal sensitization, as detailed in the text.

The respiratory tract has the most extensive surface area for bacteria and fungi to interact. Fungus-bacterium interactions are bidirectional with a variety of reciprocal interactions encompassing antagonistic and pathogenetic interactions in addition to beneficial ones (Nogueira et al., 2019; Zhang et al., 2022). Mutually beneficial interactions are also possible in mixed-biofilm environments, where the different species shield each other against an invading immune response or antimicrobial agent (Peleg et al., 2010). Thus, similar to the gut, their dynamic interaction might affect the growth and virulence of bacteria and fungi in the lung. Mechanistically, bacteria and fungi can interact in several ways, including physical interactions by direct cell–cell contact, with fungi serving as a biological scaffold for bacterial attachment, chemical interaction through the secretion of small molecules that are often involved in quorum sensing, environmental modifications such as pH changes, use of metabolic by-products and alterations in host responses (Peleg et al., 2010). A very recent study has shown that convergent evolution in toxin detection and resistance may promote conserved bacterial–fungal interactions by enhancing the fitness of both microbial species in the lung (Dolan et al., 2024). Interestingly, it has also been shown that the lung pathogens can interact at a distance via volatile-mediated communication (Briard et al., 2016). There is no doubts that the coexistence of *A. fumigatus* and *P. aeruginosa* in the lung, particularly of individuals with CF, has long been known for its association with disease exacerbation (Reece et al., 2017). However, both antagonistic and mutually beneficial interactions have been described, with *P. aeruginosa* both inhibiting (Briard et al., 2015; Sass et al., 2018; Briard et al., 2017) and promoting (Briard et al., 2016) the growth and virulence of *Aspergillus*. In retaliation, *A. fumigatus* inhibited the biofilm formation of *P. aeruginosa* through gliotoxin production (Reece et al., 2018) or iron competition (Sass et al., 2019). Ultimately, modulating the availability of iron and biotin to bacterial species has recently been found to represent conserved drivers of bacterial-fungal interactions (Pierce et al., 2021). In the presence of bacteria, such as *P. aeruginosa* and *Stenotrophomonas maltophilia*, *Aspergillus* also modifies the cell wall and hyphal morphology thus favoring bacterial sticking (Briard et al., 2017; Melloul et al., 2016). Although less frequent, the interactions of *Aspergillus* with other bacteria have also been described, such as the co-existence with *Haemophilus* in fungal ball rhinosinusitis, the main type of non-invasive fungal sinusitis, suggested to have a role in pathogenesis (Delliere et al., 2021), the antagonistic relationship with *S. aureus* in an vitro model mimicking infectious keratitis (Ramirez et al., 2015) or the synergistic pathogenetic role with *Mycobacterium abscessus* in experimental CF (Monin et al., 2018) (Figure 2). Association of *P. aeruginosa* with *Candida albicans* in the respiratory tract of individuals with CF has also been observed particularly in association with anaerobes (Delhaes et al., 2012). Because anaerobic growth of *C. albicans* may promote resistance to antifungal drugs (Dumitru et al., 2004), this may suggest an antagonistic interaction between *P. aeruginosa* and *C. albicans* in an anaerobic environment. However, both species mutually suppress biofilm development and growth, a finding highlighting the complexity of the pathobiology of mixed bacterial-fungal infections (Bandara et al., 2010).

Ultimately, with the widespread use of antibacterial and immunomodulatory drugs and increasing number of immunocompromised patients, pulmonary bacterial-fungal co-infections are becoming more common (Katsoulis et al., 2024), as suggested by a recent study in which co-infections were present in more than 40% of patients with fungal pneumonia and favored by the underlying immune status and the presence of pulmonary cavities (Zhao et al., 2021). Consistently, the altered immune status determines the interaction of fungi with bacteria in asthma, a pathological condition in which different bacterial-fungal co-occurrence patterns have been described across asthma-associated phenotypes and endotypes and correlated with clinical parameters (Sharma et al., 2019; Liu et al., 2020; Barac et al., 2018). In addition, the advanced sequencing technologies are expanding the detection of co-infections in lung pathologies such as COPD in which a significant inverse correlation between bacteria and fungi has been described in COPD patients versus controls, and in frequent versus non-frequent exacerbators. In particular, commensal bacterial taxa such as *Prevotella.* and *Veillonella* spp. exhibited inverse relationships with pathogenic fungal taxa such as *Candida palmioleophila* and *Aspergillus* spp. Importantly, the perturbed bacterial-fungal interactions in COPD were associated with increased airway inflammatory mediators such as IL-6 and IL-8 (Liu et al., 2021). Thus, the disruption of airway bacterial-fungal community balance, characterized by the loss of commensal bacterial taxa and enriched pathogenic fungal taxa, could be implicated in COPD. These few examples highlight the complex nature of fungal-bacterial interactions in the mammalian lung, consistent with the broad impact of fungi on bacterial fitness within microbiomes, as revealed by combined high-throughput genetic screening, RNA-Seq, bacterial cytological profiling, and metabolomics of bacterial-fungal interactions (Pierce et al., 2021). Not less important, is the sensitivity of the lung mycobiome and its interaction with bacteria from the gut along the gut-lung axis (Marsland et al., 2015; Katsoulis et al., 2024; Ozcam and Lynch, 2024; Enaud et al., 2020). Indeed, information exchange between these two large mucosal surface areas regulates microorganism-immune interactions, with significant implications for the clinical and treatment outcomes of a range of respiratory conditions, including asthma, chronic obstructive pulmonary disease, nosocomial pneumonia and lung cancer. Ultimately, the crucial role of the fungal-bacterial interactions across habitats and ecosystems is well established (Deveau et al., 2018).

## 5 Adding the host: the tripartite interaction

There is no doubts that the microbiota can regulate microbial homeostasis itself by preventing the growth and spread of potential harmful microorganisms and, therefore, avoiding their recognition by the immune system. However, bacteria and fungi interact with the immune system at mucosal surfaces in ways that are important for both host defence and regulation of the immune system (Katsoulis et al., 2024; Underhill and Iliev, 2014). Thus, despite constantly being in flux with rapid clearance, the lung microbiota leaves important signatures and imprints on the airway immune tone (Park et al., 2024). And indeed, in addition to antimicrobial defense provided via a number of direct and indirect mechanisms of colonization resistance (Thibeault et al., 2021), one important function of the microbiota is to promote and maintain a state of immune tolerance at mucosal surfaces, to prevent not desirable inflammatory response and mucosal damage (Mendez et al., 2019; Herbst et al., 2011). As anticipated, the promotion of tolerance to allergen challenge starts very early in the life (Gollwitzer et al., 2014) and is mediated by a continuous dialog between microbes and immune resident cells, such as epithelial cells, alveolar macrophages and dendritic cells, innate and adaptive lymphoid cells, including Tregs and specific subsets of resident memory B and T cells, all expressing a repertoire of innate sensory receptors capable to recognize molecules of host and microbial origin and to tailor the subsequent response accordingly (Wypych et al., 2019; Park et al., 2024; Natalini et al., 2023; Lipinksi et al., 2024). Their activation promotes not only the initiation of innate and adaptive immune response against pathogens but also a tonic receptor activation that is essential to preserve the epithelial barrier integrity (Park et al., 2024; Lipinksi et al., 2024; Li et al., 2024; Schreiber et al., 2024). In healthy lungs, indeed, the repeated or chronic exposure to endotoxin may cause immune and epithelial cells to acquire a tolerogenic phenotype (Biswas and Lopez-Collazo, 2009). In this regard, by providing a strong barrier, the airway epithelium serves as the primary line of defense against potentially harmful environmental irritants. However, in chronic lung diseases, increased mucus production by epithelial cells facilities the growth of specific bacteria via hypoxia and inflammation (Glover and Colgan, 2017). Although further investigations are needed to fully understand the role of innate receptors in governing respiratory mucosal functions, this signaling is generally suppressed or mitigated at mucosal surfaces through a variety of mechanisms, including receptor cooperativity and the cytokine milieu (Mendez et al., 2019).

Microbes and metabolites also influence adaptive immune responses for the maintenance of lower airway immune tone. Mouse aspiration of a mixture of oral commensals (*Prevotella melaninogenica*, *Veillonella parvula* and *Streptococcus mitis*) leads to recruitment of IL-17-producing T helper cells (Th17) and γδ T cells and counter-regulatory activation of Tregs and the immune-checkpoint inhibitor PD1 marker on T cells (Wu et al., 2021). In humans, members of the Bacteroidetes phylum decreases lung inflammation, while microbiota rich in oral-associated taxa, such as *Veillonella*, *Rothia*, *Rhodobacter* and *Prevotella* spp. (Segal et al., 2013; Segal et al., 2016) or rich in Proteobacteria families of Pasteurellaceae and Enterobacteraceae are related to Th17 cell-mediated lung inflammation and allergy (Huang et al., 2015). It has been demonstrated that Gammaproteobacteria, through their ability to utilize inflammatory byproducts to survive and propagate under low oxygen conditions, such as chronic inflammation, can outcompete bacteria that are unable to metabolize inflammatory byproducts for survival (Winter et al., 2013; Winter and Baumler, 2014; Scales et al., 2016). Thus, the shifts from Bacteroidetes dominating the healthy lung towards Gammaproteobacteria can be considered a microbial signature of a diseased lung (Huffnagle et al., 2017). As a matter of fact, it has been demonstrated that URT bacteria (*Prevotella* and *Veillonella* spp.) essentially regulate the level of airway inflammation and immune tone in the lower airways (Segal et al., 2016) thus counter-regulating inflammatory Proteobacteria.

It is now clear that microbiome-derived metabolites have mechanistic implications for lung diseases (Moitra et al., 2023). Consistently, tryptophan (Trp) locally or distally metabolized, by both the mammalian host and the microbiota into a number of metabolites (see below), is known to affect immune and microbial homeostasis in the lung (Montagnoli et al., 2006; Choera et al., 2017; Renga et al., 2023; Nunzi et al., 2024; Iannitti et al., 2013; Costantini et al., 2024; Romani et al., 2008), while products of bacterial anaerobic metabolism, like butyrate and other SCFAs, induce Tregs (Segal et al., 2017). All these studies indicate that commensal microorganisms that colonize the respiratory tract are critical in maintaining and sustaining immune “tolerance” in the lung and that the aberrant and chronic innate or adaptive immune reactivity associated with microbial dysbiosis characterizes lung pathologies, from susceptibility to infections and allergy to lung remodeling in degenerative diseases and promotion of cancer (Sommariva et al., 2020). On the other side, however, Tregs depletion by aerosolized antibiotics or probiotics supplementation could enhance lung immunosurveillance in cancer (Le Noci et al., 2018).

## 6 The host-microbial metabolism

The host organism is a complex mosaic of cell populations that requires adequate supplies of nutrients for maintenance, growth and proliferation. Because many nutrient requirements may be shared by host cells, pathogens and indigenous microbiota, all these cells may potentially compete for growth-limiting resources. Thus, metabolism is important and the microbiota may play a role in host metabolism (Costantini et al., 2024). However, bacterial metabolism by itself is known to modulate host pulmonary immunity. For instance, *E. coli* and *S. aureus*, two pathogenic bacteria in the context of the lung microbiome, have been linked to the episodic increases in proteases and protease inhibitors that play a role in infection and immune response (Davies et al., 2010). In CF, it has recently been reported that *A. fumigatus* is able to “shape the lung microbiome towards a more beneficial fungal growth environment associated with aromatic amino acid availability and the shikimate pathway” (Mirhakkak et al., 2023). Thus, pointing to the emerging role of metabolic perturbations due to microbial dysbiosis, inter-kingdom metabolism may become the key in the regulation of microbiome-pathogen cross-talk. The competition for nutrients may tips the balance towards protection, but metabolic rewiring to sustain pathogen presence and growth may become crucial in infection. Metabolomics has indeed revealed differences in lung diseases and infections compared to healthy controls and it is believed that these differences could originate from microbiome alterations (Moitra et al., 2023). Thus, metabolic phenotyping (metabotyping) may serve to identify phenotypes and endotypes of respiratory disease (Reinke et al., 2022).

As anticipated, Trp is a central hub for host/microbial information processing. Besides being involved in protein synthesis, Trp is a versatile precursor and can be metabolized by both host and microbial enzymes to generate a variety of molecules involved in different fundamental processes (Nunzi et al., 2024; Agus et al., 2018; Romani et al., 2015; Borghi et al., 2020; Li, 2023). Three pathways have gained considerable interest for their role at the interface between the host, the microbiome and pathogens. These pathways include the host kynurenine and serotonin pathways and the microbial indole pathway (Nunzi et al., 2024). The host kynurenine pathway and the microbial indole pathway, converge on a central xenobiotic receptor, the Aryl Hydrocarbon Receptor (AhR), a critical regulator of the host/microbe interface (Zelante et al., 2013; Dong and Perdew, 2020; Stockinger et al., 2024; Zimmerman et al., 2024; Guerrina et al., 2018; Puccetti et al., 2018). The kynurenine pathway accounts for ∼95% of overall Trp degradation and the first and rate-limiting step is mediated by indoleamine 2, 3–dioxygenase (IDO) 1, along with IDO2 (a paralogue of IDO1) and the tryptophan 2,3-dioxygenase, resulting in the formation of kynurenines. The kynurenine pathway, and IDO1 in particular, has been associated with the promotion of tolerance in the lung (Iannitti et al., 2013). For instance, the Trp metabolic pathway crucially provides immune homeostasis in fungal infections by taming heightened inflammatory responses and inducing immune and tissue tolerance, an activity to which the host, fungi and the microbiota cooperatively contributed (Romani, 2011). The serotonin pathway also influences the interactions between host and microbes (Nunzi et al., 2024). For instance, commensal bacteria regulate the synthesis of serotonin by the host (Yano et al., 2015), and serotonin may modulate the composition of the gut microbiome (Kwon et al., 2019). Along this direction, we have recently demonstrated an emerging role of serotonin in orchestrating host and microbial dialogue at the level of Trp utilization to the benefit of local and systemic homeostasis (Renga et al., 2023; Nunzi et al., 2024). In particular, the Trp hydroxylase (TPH) 1/5-hydroxytryptamine pathway leading to serotonin production calibrated host and microbial Trp metabolism during *A. fumigatus* pneumonia. By so doing, serotonin essentially contributed to pathogen clearance and immune homeostasis in infection by promoting the host protective IDO1/kynurenine tolerogenic pathway and limiting the indole/AhR pathway through microbial education. The third important pathway is represented by the indole pathway whereby different species of bacteria produce indole compounds via different metabolic pathways (Nunzi et al., 2024; Dong and Perdew, 2020). Indoles are very attractive molecules as they have been shown to augment health span across a broad range of evolutionarily diverse species from different phyla, to control bacterial fitness, including interspecies signaling cross-talk, antibiotic resistance and strengthening of the epithelial barrier function (Jin et al., 2014; Dvorak et al., 2020; Roager and Licht, 2018; Puccetti et al., 2023). Intriguingly, fungi possess Trp-degrading enzymes (Yuasa and Ball, 2012; Koper et al., 2022) that allow them to metabolize Trp to kynurenines as well as to indolepyruvate (Choera et al., 2017). This enzymatic repertoire allows *A*. *fumigatus* to metabolize Trp in a context-dependent manner when adapting to the host niche (Zelante et al., 2021a) and to shape the lung microbiome (Mirhakkak et al., 2023). All together, the host IDO1 and TPH1 pathways and the microbial–dependent AhR pathway, although intersecting at a common node, appear to promote distinct but complementary functions in immune and microbial homeostasis at mucosal surfaces (Renga et al., 2023; Zelante et al., 2013; Puccetti et al., 2018; Zelante et al., 2021b). In the lung, in particular, it was found that the loss of beneficial bacteria and immune tolerance at the barrier surface was associated with reduced levels of Trp metabolites, such as indole-3-aldehyde in the pharynx and kynurenine in the nose, in hematologic patients at risk for invasive aspergillosis (Costantini et al., 2022; Costantini et al., 2021).

Moving forward towards the exploitation of shared metabolism between the host and microbes for therapeutic purposes, we were intrigued by the potential important role of the enzyme sphingosine-1-phoshate lyase (SPL) catalyzing the irreversible degradation of sphingosine-1-phosphate (S1P) in regulating the sphingolipid metabolism at the host-fungus interface (Cellini et al., 2023). Consistent with the immunomodulatory activity of S1P (Veltman et al., 2016) and its antifungal activity (Rollin-Pinheiro et al., 2016), SPL inhibition proved to be of therapeutic benefit in a murine model of fungal pneumonia (Cellini et al., 2023). This study represents a proof-of-concept demonstration of the exploitation of trans-kingdom metabolic unique features for therapeutic purposes. Finally, consistent with the influence of advanced glycation endproducts (AGEs) on gut permeability through the microbiota (Snelson et al., 2022), our recent finding that a defective detoxification of the dicarbonyl methylglyoxal, one major precursor of AGEs, contributed to inflammatory pathology in CF (Pariano et al., 2021) suggests a possible influence of AGEs on epithelial permeability and microbiota in the respiratory tract.

## 7 Conclusion and future directions

Despite the recent advances in the identification of microbial signatures as potential biomarkers of illness of lung diseases and malignancies, further insights are needed to translate the bulk of knowledge into clinical practice. Very challenging, at this point, is the potential therapeutic of modulating the lung microbiome on lung diseases or to preserve microbial eubiosis during antibiotics, inhaled corticosteroids and other treatments, known to alter the respiratory microbiota (Durack et al., 2017; Hisert et al., 2017). On this regard, it is worth considering that the impact of antibiotics on the lung microbiome may have more far-reaching implications than expected. Indeed, it is given that the bacterial component of the microbiome is affected but, in turn, these changes may have repercussions on the bacteria-fungi interactions with different outcomes. Indeed, fungal overgrowth may occur in response to bacterial depletion, but opposite effects have also been demonstrated, with fungal growth reduced by expansion of antagonistic bacteria in response to antimicrobial treatment (Spatz et al., 2023). Despite the therapeutic and economical potential, microbiome therapeutics is still in the developing stage and is facing technical and administrative issues. The enormous metabolic potential of microbes and their role in the maintenance of human health is emerging, thus broadening the landscape of microbiome therapeutics. As highlighted in this review, the emerging importance of the inter-kingdom cross-talks and its impact on immune and metabolic state of the lung cannot be disregarded in microbiota-oriented future medicine.
